# The cross-regional settlement methods in hospitals and the treatment-seeking behavior of patients with malignant tumors in China: an evolutionary game model

**DOI:** 10.3389/fpubh.2024.1427164

**Published:** 2024-07-17

**Authors:** Xinzhe Zhao, Linjin Li, Dan Zhang

**Affiliations:** ^1^Genertec Universal Medical Group, Beijing, China; ^2^Institute for Hospital Management, Tsinghua Shenzhen International Graduate School, Tsinghua University, Shenzhen, China

**Keywords:** cross-regional treatment, direct settlement, malignant tumors, treatment-seeking behavior, evolutionary game

## Abstract

**Background:**

Cross-regional settlement management is a key indicator of national health insurance system maturity. Given the significant demand for cross-regional medical treatment among Chinese patients with malignant tumors and the territorially managed health insurance system, further research is necessary to explore the relationship between hospital settlement methods and treatment-seeking behaviors among these patients. This study introduces and validates an evolutionary game model that provides a theoretical foundation for direct settlement policies in cross-regional treatment.

**Methods:**

An evolutionary game model was constructed with patients and hospitals serving as strategic players within a dynamic system. This model integrates the patients’ treatment utility, medical and nonmedical costs, and hospitals’ financial and technological advancement benefits.

**Results:**

The evolutionary stability analysis revealed seven-game outcomes between hospitals and patients with malignant tumors. The numerical simulations suggest an evolutionary convergence toward strategy (1, 0), indicating a trend where patients with malignant tumors opt for cross-regional treatment, yet hospitals choose not to implement a direct settlement policy. Parameter sensitivity analysis showed that the parameters set in this study affected player behavioral choices and game equilibria.

**Conclusion:**

A strong demand for cross-regional medical treatment among Chinese patients with malignant tumors, and some hospitals require more incentives to implement cross-regional settlements. The key factors influencing the willingness of some patients with malignant tumors to resettle include the costs of in-area medical care, costs of cross-regional treatment without direct settlement, and the utility of cross-regional treatment. Technological advancement benefits and input costs influence some hospitals’ motivation to adopt cross-regional settlements. Policy adjustments that effectively implement direct settlement policies can facilitate equilibrium, enhance the initiatives of some local health insurance management departments, improve the accessibility and efficiency of medical services, and reduce nonmedical expenses for patients.

## 1 Introduction

Cross-regional treatment refers to the act of an insured individual seeking medical care in areas outside their insurance coverage under the healthcare security system (1–3). The uneven geographical distribution of healthcare resources is a major factor driving the increase in the number of patients opting for medical services in various locations (4). The scope of insurance coverage varies between insured and uninsured areas, with the insured areas encompassing entities such as countries, cities, and other administrative regions (5). For instance, within the European Union (EU), each member state typically has its own insurance coverage zone. To improve citizens’ access to healthcare, the EU offers the European Health Insurance Card, which allows nationals from member states to receive medical services across the union. The variability in healthcare policies among countries necessitates full harmonization of medical insurance payments and reimbursement standards. The EU responded by implementing Directive 2011/24/EU of the European Parliament and Council on the application of patients’ rights in cross-border healthcare, ensuring patients’ rights to receive medical services and insurance reimbursements in other EU countries (6).

Due to China’s household registration system, cross-regional treatment is divided into unplanned and planned categories. The unplanned category typically includes short-term business travelers, migrant workers, and retired residents relocating to different regions, whereas the planned category is driven by individuals seeking superior healthcare services due to the unequal distribution of medical resources (7, 8). This study is focused on planned cross-regional settlements. Unlike the EU, China’s medical insurance pooling is limited to the city level, leading to cross-regional settlement reimbursements for patients (9). With this flexible health insurance management mechanism, China has achieved a medical insurance coverage rate of over 95% for its 1.4 billion population, approaching the threshold of universal health coverage (10). Although significant progress has been made in health insurance coverage, it has also introduced a unique challenge: the issue of cross-regional settlement (11). Residents seeking medical care outside their insured areas encounter obstacles related to inconsistent financing and payment standards, which are often exacerbated by complex reimbursement processes. In response, China has implemented policies for cross-regional settlements. In 2020, the Chinese government issued “Opinions on Deepening the Reform of the Medical Security System,” proposing further improvements in the direct settlement system for cross-regional medical treatment (12). In July 2022, the Chinese government issued the “Notice on Further Improving the Direct Settlement of Basic Medical Insurance for Cross-Province Medical Treatment,” aiming to increase the direct settlement rate of inpatient expenses by over 70% by 2025 (13). The direct settlement policy for cross-regional medical treatment meets the needs of non-insured residents to some extent. In the first three quarters of 2023, China had 84.54 million cross-province direct settlement cases for medical treatment, reducing individual out-of-pocket expenses by 15.5 billion dollars (14).

Patients with malignant tumors in China require direct settlement for cross-regional medical treatment. Regardless of economic status across countries, malignant tumors are one of the most common causes (15). In China, malignant tumors rank second in the mortality rate of resident diseases (16), with an annual increase of 21.6% in deaths from these tumors (2005–2020) (17). Due to the concentration of high-quality resources for cancer diagnosis and treatment, among patients undergoing cross-regional treatment, those with malignant tumors constitute one of the largest groups (2, 5, 11). A Research shows that from 2015 to 2020, chemotherapy for tumors and lung cancer ranked first and fifth, respectively, among cross-regional treatments in China. Even in 2020, a year after the COVID-19 pandemic, the proportion of patients traveling for chemotherapy continued to rise (18). However, an imperfect real-time settlement system for cross-regional medical treatment often requires patients to make advance payments, adding a considerable financial burden on families (19). Lengthy reimbursement cycles and complex travel arrangements exacerbate this problem, adversely affecting the daily lives of patients and their families (20). Moreover, the frequent visits and follow-ups required for treating malignant tumors further intensify the economic strain on families (21).

In cross-regional direct settlement issues in China, hospitals and patients with malignant tumors have different considerations. The primary objective of hospitals is to enhance their technical capabilities and service quality to achieve sustainable development (22). By implementing direct cross-regional settlement, hospitals can attract more patients, thus continuously optimizing and improving their expertise. When treating cross-regional patients, hospitals often adopt a fee-for-service approach that allows them to generate more financial revenue (23). In 2023, the number of hospitals in China implementing direct settlement for inpatient expenses increased by 19,600 from the previous year, indicating that despite the widespread implementation of the policy, not all institutions have adopted this service (24). One significant challenge is the information technology transformation of medical insurance platforms, which incurs substantial maintenance costs (25). Additionally, hospitals face risks associated with higher upfront financial payments and longer reimbursement cycles (26). For patients with malignant tumors, focusing on securing improved diagnoses and treatment outcomes through direct cross-regional settlement while reducing the healthcare burden resulting from inconvenient medical insurance in their local areas is essential (23). Since oncological treatment resources are primarily concentrated in first-tier cities and provincial capitals, patients with malignant tumors often opt to seek medical care in these areas to access more specialized expertise and enhanced treatment outcomes (17). Importantly, the settlement method for cross-regional medical treatment matches the medical insurance coverage of the treatment location, following the specific drug formulary, diagnostic procedure list, and medical consumables catalog (27). The costs of services not listed in these catalogs are the patient’s responsibility and are not covered by insurance. These locations typically offer more comprehensive insurance catalogs, allowing patients to receive higher reimbursements and reduce their out-of-pocket expenses (28). Patients can also significantly reduce the time and effort required to process medical insurance reimbursements (11).

Evolutionary Game Theory was first introduced by John Maynard Smith and George R. Price in their seminal paper “The Logic of Animal Conflict,” published in the journal Nature, applying Darwinian principles to the study of strategic interactions within populations (29, 30). It applies mathematical principles to predict and understand behavior in a variety of complex systems ranging from animal dynamics to human social and economic interactions (31). It is increasingly applied across various fields, such as mathematics, economics, and business management (32). Its extension to the field of healthcare management is also noteworthy, encompassing areas such as healthcare reforms (33), healthcare and social care coordination (34), and early warning of public health emergencies (35).

In this study, Evolutionary Game Theory has several advantages. Initially, it broadened the scope of analysis from individuals to groups, increasing its relevance and applicability (35). Second, it employs population thinking, emphasizing heterogeneity within groups and allowing for individual strategies within the same group (36). Third, it emphasizes the importance of the historical context and frequency of strategies (31, 37), which helps understand the evolution of behaviors within patient and hospital groups over time. Furthermore, despite data challenges such as regional dispersion and privacy in China (18), it can explore the interactive behaviors between hospitals and patients and conduct sensitivity analyses to test outcomes under various parameters, even without complete data. Finally, it assumes limited rationality, reflecting more realistic decision-making processes based on partial information and limited decision capabilities and aligning closely with the practicalities of real-world scenarios (38).

In the field of public health, research on cross-regional medical treatments for patients with malignant tumors has primarily focused on mobility (15, 39), service utilization (3, 40), and influencing factors (2, 5, 11). These studies overlooked the strategic interactions between patients and healthcare providers, as well as the significant impact of direct settlement policies. Therefore, this study uses Evolutionary Game Theory to provide new insights into the relationship between the behaviors of patients with malignant tumors and hospital policies on cross-regional settlement, aiming to fill the gaps left by previous research. Compared with international practices, China lacks policy support for the settlement of cross-regional medical treatments for patients (41). This study aimed to uncover the group evolutionary paths of patients with malignant tumors and hospitals in the absence of policy influence, providing a theoretical foundation for policy development.

In the subsequent sections of this study, Section 2 presents the parameters and assumptions of the evolutionary game model. Section 3 presents the construction of the model and analysis of the equilibrium of the system. Section 4 presents the numerical simulations. Section 5 discusses the findings, provides the theoretical and policy implications, and highlights the limitations of the study. Finally, Section 6 presents the conclusions drawn from the study.

## 2 Model design

### 2.1 Description of players

In the implementation of the direct settlement policy for cross-regional medical treatment, the primary stakeholders are hospitals and patients with malignant tumors. Both groups, considered as limited rational players, continually adapted their strategies based on ongoing interactions, ultimately aiming for a stable system equilibrium. The strategic combinations of patients with malignant tumors and hospitals are listed in Table 1.

**Table 1:** Strategic combinations of patients with malignant tumors and hospitals.

Strategies		Hospitals	
		Direct settlement for cross-regional medical treatment	Indirect settlement for cross-regional medical treatment
Patients with malignant tumors	Cross-regional treatment	Patients seek cross-regional treatment, hospitals’ direct settlement	Patients seek cross-regional treatment, hospitals’ indirect settlement
	In-area medical care	Patients seek in-area medical care, hospitals’ direct settlement	Patients seek in-area medical care, hospitals’ indirect settlement

In this study, “hospitals” refer to hospitals that have the autonomy to decide whether to implement the direct settlement policy. Considering the municipal-level management characteristics of China’s medical insurance policy, a direct settlement policy has regional features (42). For example, in Anhui Province, hospitals can independently choose whether to adopt a direct settlement policy by submitting an application to the Provincial Cross-regional Medical Treatment Management Center (43). However, not all scenarios are considered, and a considerable range of real-world settings are represented. It assumes that hospitals decide between “direct settlement for cross-regional medical treatment” and “indirect settlement.”

This study focused on patients with malignant tumors undergoing planned cross-regional medical treatment. This includes individuals diagnosed with cancer types that present greater clinical challenges and poorer prognoses, such as pancreatic cancer. In contrast, patients with malignant tumors that present less clinical difficulty and have better prognoses, such as thyroid cancer, were not included in this study. It assumes patients with malignant tumors decide between “cross-regional treatment” or “in-area medical care.”

### 2.2 Model assumptions and parameters setting

In the assumptions of this model, the policy for direct settlement of cross-regional medical treatment includes three characteristics that align with actual conditions. Firstly, regarding policy coverage, it is presumed that all provinces nationwide have implemented the direct settlement policy, though not all hospitals proactively offer this service, reflecting real-world practices (44). Secondly, concerning reimbursement ratios, the model assumes that the reimbursement rate for malignant tumor patients seeking treatment outside their insured region exceeds that within their insured location under equivalent treatment conditions. As previously mentioned, this study focuses on unplanned cross-regional medical treatments undertaken by patients in pursuit of superior medical resources; It has been validated that these areas typically have more comprehensive medical insurance catalogs, thereby affording higher reimbursement rates under the same treatment conditions (27). Thirdly, regarding the direct settlement reimbursement process, the model assumes uniformity across provinces: patients use the national medical insurance service platform’s APP or the WeChat Mini Program of the National Medical Insurance Administration to apply for cross-provincial medical treatments online and facilitate real-time settlements (45).

Besides, the impacts of COVID-19 or similar public health emergencies are not considered. During the pandemic, the localized management of health policies in China temporarily restricted cross-regional medical treatment (46), which returned to pre-pandemic conditions post-COVID-19. Given the intensified focus on healthcare in the post-pandemic era and the enhanced mobility of the population, there is a potential increase in the demand for cross-regional medical services (47). Therefore, the analysis and conclusions of this study are based on conditions free from such external disruptions, further underscoring the practical significance of our study in a non-pandemic context.

The model assumptions for patients with malignant tumors are presented in Table 2. In the assumptions, x represents the probability of choosing cross-regional treatment, whereas 1−x indicates the probability of choosing in-area medical care, where 0≤x≤1 . Medical services are utilized through treatment methods and approaches that improve patient health (48). The utility derived from cross-regional medical care is denoted by $U_{1}$ , whereas $U_{2}$ represents the utility obtained from in-area medical care, where $U_{1}$ > $U_{2}$ . Furthermore, studies have indicated that under direct settlement policies, patients with malignant tumors who seek cross-regional treatment incur lower out-of-pocket expenses than those receiving in-area medical care when using the same treatment methods (28). Thus, $S_{1}$ signifies the medical costs incurred by patients for cross-regional treatment, whereas $S_{2}$ represents the cost of in-area medical care, where $S_{1}$ < $S_{2}$ . Additionally, $C_{1}$ encompasses other costs associated with cross-regional treatment and direct settlement, such as travel, accommodation, and time-related expenses, whereas $C_{2}$ signifies equivalent costs without direct settlement, where $C_{1}$ < $C_{2}$ .

**Table 2:** Model assumptions for patients with malignant tumors.

Case	Parameters	Definition	Ranges
Cross-regional treatment	x	Probability of choosing cross-regional treatment	0≤x≤1
	$U_{1}$	Utility of cross-regional treatment	$U_{1}>U_{2}$
	$S_{1}$	Medical costs paid by patients after cross-regional treatment	$S_{1}<S_{2}$
	$C_{1}$	Other costs of cross-regional treatment with direct settlement	$C_{1}<C_{2}$
	$C_{2}$	Other costs of cross-regional treatment without direct settlement	$C_{1}<C_{2}$
In-area medical care	1−x	Probability of choosing in-area medical care	0≤y≤1
	$U_{2}$	Utility of in-area medical care	$U_{1}>U_{2}$
	$S_{2}$	Medical costs paid by patients after in-area medical care	$S_{1}<S_{2}$

The model assumptions for hospitals are presented in Table 3. In the assumptions, y represents the probability of a hospital choosing a direct settlement for cross-regional treatment, whereas 1−y represents the probability of not choosing a direct settlement, where 0≤y≤1 . $\pi_{1}$ and $\pi_{2}$ represent the financial and technological advancement benefits, respectively, of choosing a direct settlement for cross-regional treatment. Hospitals that choose direct settlement can attract more cross-regional patients, who often have more severe conditions and higher medical expenses (23). Consequently, $\pi_{3}$ denotes the financial benefit of not implementing a direct settlement, with $\pi_{1}>\pi_{3}$ and $\pi_{2}>0$ . $C_{3}$ represents the input costs for direct settlement, including fees for updating the direct settlement information system, payments related to capital turnover pressure, and delayed reimbursements, where $C_{3}>0$ .

**Table 3:** Model assumptions for hospitals.

Case	Parameters	Definition	Ranges
Direct settlement for cross-regional medical treatment	y	Probability of implementing direct settlement for cross-regional treatment	0≤y≤1
	$\pi_{1}$	Financial benefits of implementing direct settlement for cross-regional treatment	$\pi_{1}>\pi_{3}$
	$\pi_{2}$	Technological advancement benefits of implementing direct settlement for cross-regional treatment	$\pi_{2}>0$
	$C_{3}$	Input costs of implementing direct settlement for cross-regional treatment	$C_{3}>0$
Indirect settlement for cross-regional medical treatment	1−y	Probability of implementing indirect settlement for cross-regional treatment	0≤y≤1
	$\pi_{3}$	Financial benefits of implementing direct settlement for local treatment	$\pi_{1}>\pi_{3}$

Based on the parameter assumptions in Tables 2, 3, a Game Payoff Matrix was constructed for patients with malignant tumors and hospitals, as shown in Table 4.

**Table 4:** Payoff matrix between patients with malignant tumors and hospitals.

Strategies		Hospitals	
		Direct settlement for cross-regional medical treatment	Indirect settlement for cross-regional medical treatment
Patients with malignant tumors	Cross-regional treatment	$U_{1}-S_{1}-C_{1}$ $\pi_{1}+\pi_{2}-C_{3}$	$U_{1}-S_{1}-C_{2}$ $\pi_{3}$
	In-area medical care	$U_{2}-S_{2}$ $-C_{3}$	$U_{2}-S_{2}$ 0

## 3 Model analysis

### 3.1 Establishment of the payoff model

The Replicator Dynamic Equation is a dynamic differential equation describing the frequency or prevalence of a group strategy (49). The expected and average payoffs for patients with malignant tumors, denoted P , choosing the cross-regional and in-area medical care strategies are $E_{P_{1}}$ , $E_{P_{2}}$ , and $E_{P}$ , respectively.

Based on Table 4, the expected payoff for patients with malignant tumors who choose the cross-regional treatment strategy is denoted as $E_{P_{1}}$ , as shown in Equation 1.

(1) $$E_{P_{1}}=y\left(U_{1}-S_{1}-C_{1}\right)+\left(1-y\right)\left(U_{1}-S_{1}-C_{2}\right)$$

The expected payoff for patients with malignant tumors choosing in-area medical care is denoted as $E_{P_{2}}$ , as shown in Equation 2.

(2) $$E_{P_{2}}=y\left(U_{2}-S_{2}\right)+\left(1-y\right)\left(U_{2}-S_{2}\right)$$

The expected payoff for patients with malignant tumors who adopt the mixed strategy is $E_{P}$ , as shown in Equation 3.

(3) $$E_{P}=xE_{P_{1}}+\left(1-x\right)E_{P_{2}}$$

The Replicator Dynamic Equation for patients choosing a cross-regional treatment is $F\left(x\right)$ , as shown in Equation 4.

(4) $$\begin{array}{l} F\left(x\right)=\frac{dx}{dt}=x\left(E_{P_{1}}-E_{P}\right)=x\left(1-x\right)\left(E_{P_{1}}-E_{P_{2}}\right)\\ =x*\left(x-1\right)*\left(C_{2}+S_{1}-S_{2}-U_{1}+U_{2}+C_{1}*y-C_{2}*y\right) \end{array}$$

Similarly, the expected and average payoffs for hospitals, denoted, that choose direct and indirect settlement strategies for cross-regional settlements are $E_{H_{1}}$, $E_{H_{2}}$ and $E_{H}$ respectively.

Based on Table 4, the expected payoff for hospitals that choose direct settlement for cross-regional treatment is denoted as $E_{H_{1}}$ , as shown in Equation 5.

(5) $$E_{H_{1}}=x\left(\pi_{1}+\pi_{2}-C_{3}\right)+\left(1-x\right)\left(-C_{3}\right)$$

The expected payoff for hospitals that choose indirect settlement for cross-regional treatment is $E_{H_{2}}$ , as shown in Equation 6.

(6) $$E_{H_{2}}=x\left(\pi_{3}\right)+\left(1-x\right)\left(0\right)$$

The expected payoff for hospitals adopting a mixed strategy is $E_{H}$ , as shown in Equation 7.

(7) $$E_{H}=yE_{H_{1}}+\left(1-y\right)E_{H_{2}}$$

The Replicator Dynamic Equation for hospitals that opt for direct settlement in cross-regional treatments is represented by $F\left(x\right)$ , as shown in Equation 8.

(8) $$\begin{array}{l} F\left(y\right)=\frac{\mathrm{dy}}{dt}=y\left(E_{H_{1}}-E_{B}\right)=y\left(1-y\right)\left(E_{H_{1}}-E_{H_{2}}\right)\\ =y*\left(y-1\right)*\left(C_{3}-\pi_{1}*x-\pi_{2}*x+\pi_{3}*x\right) \end{array}$$

The set of Replicator Dynamic Equations comprising Equations 4 and 8 is given in Equation 9.

(9) $$\left\{ \begin{array}{l} F\left(x\right)=x*\left(x-1\right)*\left(C_{2}+S_{1}-S_{2}-U_{1}+U_{2}+C_{1}*y-C_{2}*y\right)\\ F\left(y\right)=y*\left(y-1\right)*\left(C_{3}-\pi_{1}*x-\pi_{2}*x+\pi_{3}*x\right) \end{array}\right.$$

### 3.2 Evolutionary system equilibrium analysis

Setting Equation 9 to zero results in five equilibrium points:

$$O\left(0,0\right),\;A\left(1,0\right),\;B\left(0,1\right),\;C\left(1,1\right),\;D\left(x_{0},y_{0}\right)$$

$x_{0}=\frac{C_{3}}{\pi_{1}+\pi_{2}-\pi_{3}}$ , with $0<x_{0}<1$ ,

$y_{0}=\frac{C_{2}+S_{1}-S_{2}-U_{1}+U_{2}\;}{C_{2}-C_{1}}$ , with 0 $<y_{0}<1.$

An Evolutionarily Stable Strategy (ESS) is defined as the equilibrium point at which the local dynamics of the replicator dynamic equilibrium point gradually converge to a stable point (50). The evolutionary stability of an ESS can be determined using the determinant and trace of the Jacobian matrix. From Equation 9, the Jacobian matrix J can be obtained as shown in Equation 10. Let the determinant and trace of matrix J be denoted as $\det\left(J\right)$ and $tr\left(J\right)$ , respectively. When an equilibrium point satisfies $\det\left(J\right)>0$ and $tr\left(J\right)<0$ , that is, $\frac{\partial F\left(x\right)}{\partial x}<0$ and $\frac{\partial F\left(y\right)}{\partial y}<0$ , then it is an evolutionarily stable strategy in the replicator dynamics system.

(10) $$J=\left[\begin{array}{cc} \frac{\partial F\left(x\right)}{\partial x} & \frac{\partial F\left(x\right)}{\partial y}\\ \frac{\partial F\left(y\right)}{\partial x} & \frac{\partial F\left(y\right)}{\partial y} \end{array}\right]$$

The values of $\frac{\partial F\left(x\right)}{\partial x}$ , $\frac{\partial F\left(x\right)}{\partial y}$ , $\frac{\partial F\left(y\right)}{\partial x}$ and $\frac{\partial F\left(y\right)}{\partial y}$ are shown in Equations 11–14.

(11) $$\frac{\partial F\left(x\right)}{\partial x}=\left(2x-1\right)*\left(C_{2}+S_{1}-S_{2}-U_{1}+U_{2}+C_{1}*y-C_{2}*y\right)$$

(12) $$\frac{\partial F\left(x\right)}{\partial y}=x*\left(x-1\right)*\;\left(C_{1}-C_{2}\right)$$

(13) $$\frac{\partial F\left(y\right)}{\partial x}=y*\left(y-1\right)*\left(\pi_{1}-\pi_{2}-\pi_{3}\right)$$

(14) $$\frac{\partial F\left(y\right)}{\partial y}=\left(2y-1\right)*\left(C_{3}-\pi_{1}*x-\pi_{2}*x+\pi_{3}*x\right)$$

### 3.3 Evolutionary result analysis

Based on these calculations, the determinants det(J) and traces tr(J) for each equilibrium point are listed in Table 5.

**Table 5:** Analysis results of local stability.

Equilibrium	$\det\left(J\right)$	$Tr\left(J\right)$
$O\left(0,0\right)$	$\left(S_{2}-S_{1}-C_{2}+U_{1}-U_{2}\right)\left(-C_{3}\right)$	$S_{2}-S_{1}-C_{2}+U_{1}-U_{2}-C_{3}$
$A\left(1,0\right)$	$\left(C_{2}+S_{1}-S_{2}-U_{1}+U_{2}\right)\left(\pi_{1}-C_{3}+\pi_{2}-\pi_{3}\right)$	$C_{2}+S_{1}-S_{2}-U_{1}+U_{2}+\pi_{1}-C_{3}+\pi_{2}-\pi_{3}$
$B\left(0,1\right)$	$\left(S_{2}-S_{1}-C_{1}+U_{1}-U_{2}\right)*C_{3}$	$S_{2}-S_{1}-C_{1}+U_{1}-U_{2}+C_{3}$
$C\left(1,1\right)$	$\left(C_{1}+S_{1}-S_{2}-U_{1}+U_{2}\right)\left(C_{3}-\pi_{1}-\pi_{2}+\pi_{3}\right)$	$C_{1}+S_{1}-S_{2}-U_{1}+U_{2}+C_{3}-\pi_{1}-\pi_{2}+\pi_{3}$
$D\left(x_{0},y_{0}\right)$	No need to calculate	0

If the trace of the Jacobian at the equilibrium point is zero, it is not an ESS. Since tr(J) of $D\left(x_{0},y_{0}\right)$ is 0, it is not an ESS. As shown in Table 6, there are seven scenarios in the replicator system.

**Table 6:** Stability analysis of evolutionary strategic portfolios.

Scenarioc	Requirements for establishment	Equilibrium point	$\det\left(J\right)$	$Tr\left(J\right)$	Stability
1	$S_{2}-S_{1}-C_{2}+U_{1}-U_{2}<0$ $\pi_{1}-C_{3}+\pi_{2}-\pi_{3}<0$ $C_{1}+S_{1}-S_{2}-U_{1}+U_{2}>0$	$\left(0,0\right)$	+	−	ESS
		$\left(1,0\right)$	−	Uncertain	Saddle point
		$\left(0,1\right)$	−	Uncertain	Saddle point
		$\left(1,1\right)$	+	+	Unstable
2	$S_{2}-S_{1}-C_{2}+U_{1}-U_{2}<0$ $\pi_{1}-C_{3}+\pi_{2}-\pi_{3}<0$ $C_{1}+S_{1}-S_{2}-U_{1}+U_{2}<0$	$\left(0,0\right)$	+	−	ESS
		$\left(1,0\right)$	−	Uncertain	Saddle point
		$\left(0,1\right)$	+	+	Unstable
		$\left(1,1\right)$	−	Uncertain	Saddle point
3	$S_{2}-S_{1}-C_{2}+U_{1}-U_{2}<0$ $\pi_{1}-C_{3}+\pi_{2}-\pi_{3}>0$ $C_{1}+S_{1}-S_{2}-U_{1}+U_{2}>0$	$\left(0,0\right)$	+	−	ESS
		$\left(1,0\right)$	+	+	Unstable
		$\left(0,1\right)$	−	Uncertain	Saddle point
		$\left(1,1\right)$	−	Uncertain	Saddle point
4	$S_{2}-S_{1}-C_{2}+U_{1}-U_{2}<0$ $\pi_{1}-C_{3}+\pi_{2}-\pi_{3}>0$ $C_{1}+S_{1}-S_{2}-U_{1}+U_{2}<0$	$\left(0,0\right)$	+	−	ESS
		$\left(1,0\right)$	+	+	Unstable
		$\left(0,1\right)$	+	+	Unstable
		$\left(1,1\right)$	+	−	ESS
5	$C_{2}+S_{1}-S_{2}-U_{1}+U_{2}<0$ $\pi_{1}-C_{3}+\pi_{2}-\pi_{3}<0$ $C_{1}+S_{1}-S_{2}-U_{1}+U_{2}>0$	$\left(0,0\right)$	−	Uncertain	Saddle point
		$\left(1,0\right)$	+	−	ESS
		$\left(0,1\right)$	−	Uncertain	Saddle point
		$\left(1,1\right)$	+	+	Unstable
6	$C_{2}+S_{1}-S_{2}-U_{1}+U_{2}<0$ $\pi_{1}-C_{3}+\pi_{2}-\pi_{3}<0$ $C_{1}+S_{1}-S_{2}-U_{1}+U_{2}<0$	$\left(0,0\right)$	−	Uncertain	Saddle point
		$\left(1,0\right)$	+	−	ESS
		$\left(0,1\right)$	+	+	Unstable
		$\left(1,1\right)$	−	Uncertain	Saddle point
7	$C_{1}+S_{1}-S_{2}-U_{1}+U_{2}<0$ $C_{3}-\pi_{1}-\pi_{2}+\pi_{3}<0$ $S_{2}-S_{1}+U_{1}-U_{2}-C_{2}>0$	$\left(0,0\right)$	−	Uncertain	Saddle point
		$\left(1,0\right)$	−	Uncertain	Saddle point
		$\left(0,1\right)$	+	+	Unstable
		$\left(1,1\right)$	+	−	ESS

**Figure 1:**
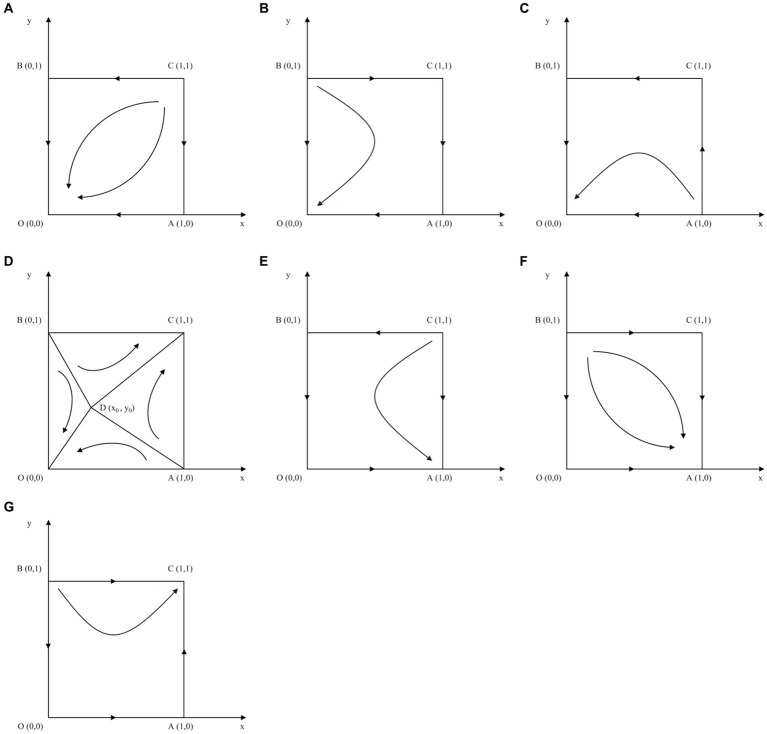
Phase diagram for the scenarios. **(A)** Scenario 1. **(B)** Scenario 2. **(C)** Scenario 3. **(D)** Scenario 4. **(E)** Scenario 5. **(F)** Scenario 6. **(G)** Scenario 7.

In Scenario 1, if $S_{2}-S_{1}-C_{2}+U_{1}-U_{2}<0$ and $\pi_{1}-C_{3}+\pi_{2}-\pi_{3}<0$ , with $C_{1}+S_{1}-S_{2}-U_{1}+U_{2}>0$ , for patients with malignant tumors, their benefits under the direct settlement for cross-regional medical treatment are greater than those for in-area medical care, whereas their benefits under the indirect settlement for cross-regional medical treatment are less than those in the insured area. Hospitals gain less from direct settlements than they do not implement them. The stable strategy is $\left(0,0\right)$ , in which patients avoid cross-regional treatment, and hospitals choose not to implement the direct settlement policy. The evolutionary process is illustrated in Figure 1A.

In Scenario 2, if $S_{2}-S_{1}-C_{2}+U_{1}-U_{2}<0$ , and $\pi_{1}-C_{3}+\pi_{2}-\pi_{3}<0$ , with $C_{1}+S_{1}-S_{2}-U_{1}+U_{2}<0$ , for patients with malignant tumors, the benefits under the direct settlement for cross-regional medical treatment are greater than those for in-area medical care. The benefits of indirect settlement for cross-regional medical treatment are more significant than those for insured areas. The stable strategy is $\left(0,0\right)$ , in which patients avoid cross-regional treatment and hospitals choose not to implement the direct settlement policy. The evolutionary process is illustrated in Figure 1B.

In Scenario 3, if $S_{2}-S_{1}-C_{2}+U_{1}-U_{2}<0$ , and $\pi_{1}-C_{3}+\pi_{2}-\pi_{3}>0$ , with $C_{1}+S_{1}-S_{2}-U_{1}+U_{2}>0$ , for patients with malignant tumors, the benefits under the direct settlement for cross-regional medical treatment are greater than those for in-area medical care. By contrast, their benefits under indirect settlement for cross-regional medical treatment are lower than those in their insured areas. The stable strategy is $\left(0,0\right)$ , in which patients avoid cross-regional treatment and hospitals choose not to implement the direct settlement policy. The evolutionary process is illustrated in Figure 1C.

In Scenario 4, if $S_{2}-S_{1}-C_{2}+U_{1}-U_{2}<0$ , and $\pi_{1}-C_{3}+\pi_{2}-\pi_{3}>0$ , with $C_{1}+S_{1}-S_{2}-U_{1}+U_{2}<0$ , for patients with malignant tumors, the benefits under the direct settlement for cross-regional medical treatment are greater than those for in-area medical care, and the benefits under the indirect settlement for cross-regional medical treatment are more significant than those in their insured area. The stable strategies are $\left(0,0\right)$ and $\left(1,1\right)$ , indicating that patients avoid or choose cross-regional treatment and hospitals opt against or for implementing the direct settlement policy, respectively. The evolutionary process is illustrated in Figure 1D.

In Scenario 5, if $C_{2}+S_{1}-S_{2}-U_{1}+U_{2}<0$ , and $\pi_{1}-C_{3}+\pi_{2}-\pi_{3}<0$ , with $C_{1}+S_{1}-S_{2}-U_{1}+U_{2}>0$ , for patients with malignant tumors, their benefits under the direct settlement for cross-regional medical treatment are less than those for in-area medical care, and their benefits under the indirect settlement for cross-regional medical treatment are less than those in their insured area. The stable strategy is $\left(1,0\right)$ , in which patients choose cross-regional treatment and hospitals opt not to implement the direct settlement policy. The evolutionary process is illustrated in Figure 1E.

In Scenario 6, if $C_{2}+S_{1}-S_{2}-U_{1}+U_{2}<0$ , and $\pi_{1}-C_{3}+\pi_{2}-\pi_{3}<0$ , with $C_{1}+S_{1}-S_{2}-U_{1}+U_{2}<0$ , for patients with malignant tumors, their benefits under the direct settlement for cross-regional medical treatment are less than those for in-area medical care, while their benefits under the indirect settlement for cross-regional medical treatment are greater than those in their insured area. Hospitals gain less from direct settlements. The stable strategy is $\left(1,0\right)$ , in which patients choose cross-regional treatment and hospitals opt not to implement a direct settlement policy. The evolutionary process is illustrated in Figure 1F.

In Scenario 7, if $C_{1}+S_{1}-S_{2}-U_{1}+U_{2}<0$ , and $C_{3}-\pi_{1}-\pi_{2}+\pi_{3}<0$ , with $S_{2}-S_{1}+U_{1}-U_{2}-C_{2}>0$ , for patients with malignant tumors, the benefits they receive under indirect settlement for cross-regional medical treatment are greater than those in their insured area. Moreover, the gap between the utility derived from cross-regional medical treatment and associated out-of-pocket medical expenses is larger than the analogous gap observed when receiving medical care in the insured region. Hospitals benefit less from a direct settlement. The stable strategy is $\left(1,1\right)$ , meaning that patients choose cross-regional treatment, and hospitals implement a direct settlement policy. The evolutionary process is illustrated in Figure 1G.

## 4 Numerical simulations

### 4.1 Evolutionary stabilization strategy

Numerical simulations were conducted using MATLAB to study the strategic dynamics between patients with malignant tumors and hospitals. The parameters were set based on prior research hypotheses and relevant research. Following the assumptions of $\pi_{1}>\pi_{3}$ and $\pi_{2}>0$ , the values for patient medical cost coefficients were based on the research of Fu (51), Han (52), and Shang (53), leading to $\pi_{1}=7$ , $\pi_{2}=2$ , and $\pi_{3}=6$ . Cost coefficients $C_{1}=$ 0.5, $C_{2}=1$ , and $C_{3}=4$ referring to Xie (26) and Li (54) were based on the assumptions $C_{1}<C_{2}$ and $C_{3}>0$ . Patient medical costs $S_{1}=3$ and $S_{2}$ =4 were assigned following the assumption $S_{1}<S_{2}$ and the findings of Wu (25) and Zheng (55). Finally, the utility coefficients $U_{1}=6$ and $U_{2}=4$ were determined based on Chen (56) and Gao (57), adhering to the hypothesis $U_{1}>U_{2}$ .

**Figure 2:**
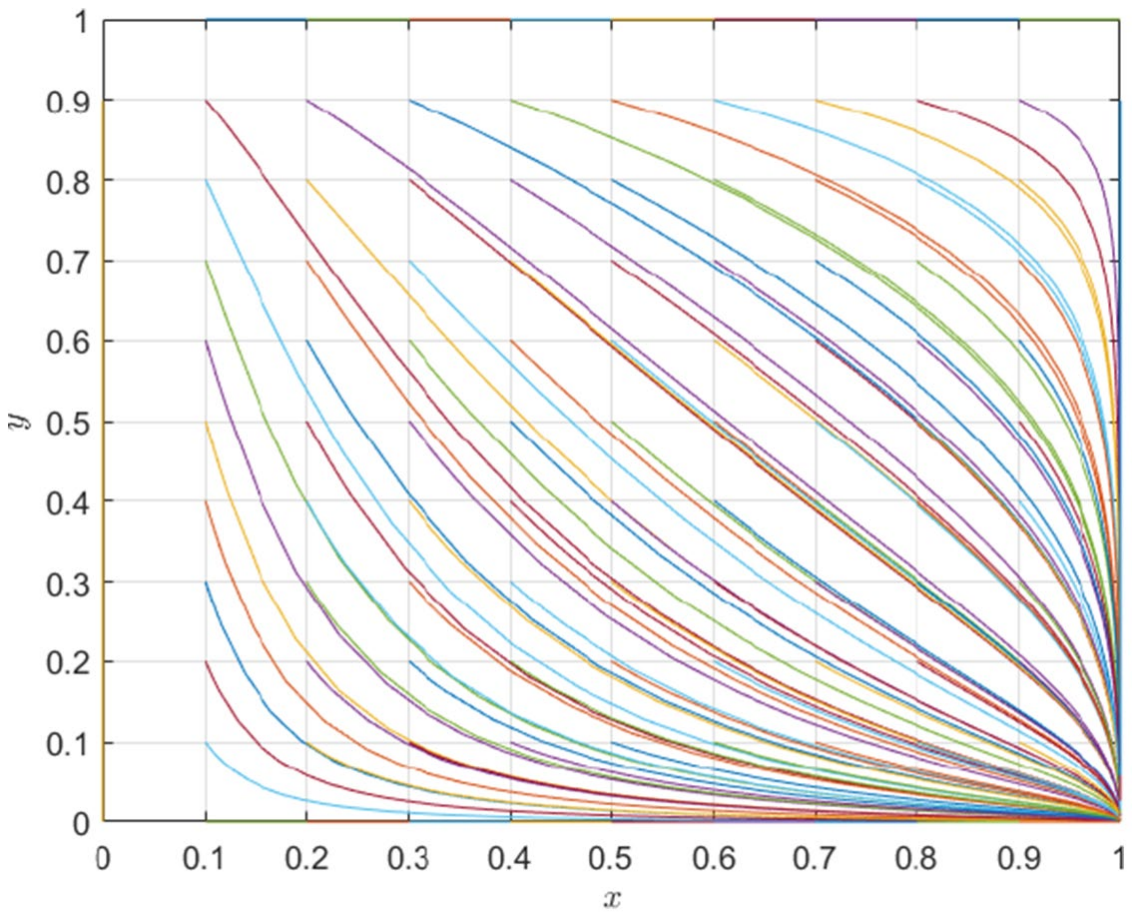
Simulation of parameters of the equilibrium point.

To ensure the generalizability of the numerical simulation findings, we used a comprehensive spectrum of simulations spanning a range of parameters from 0.1 to 1, progressing in increments of 0.1 (58). The analysis indicated an emerging scenario in which patients with malignant tumors choose cross-regional treatment, whereas hospitals tend not to adopt a direct settlement policy. This aligns with Scenarios 5 and 6 of the evolutionary game, leading to evolutionary convergence toward strategy $\left(1,0\right)$ . A simulation-based analysis conducted with varying parameters corroborated the sixth scenario of the game. Additionally, the evolution rate varied with different initial values, but the outcome remained unchanged, indicating that the change in the initial values did not affect the stability determination condition. As shown in Figure 2, the optimal strategy for patients with malignant tumors is “cross-regional treatment,” while the optimal strategy for hospitals is “indirect settlement.”

### 4.2 Parameter sensitivity analysis

#### 4.2.1 The impact of patient medical utility

**Figure 3:**
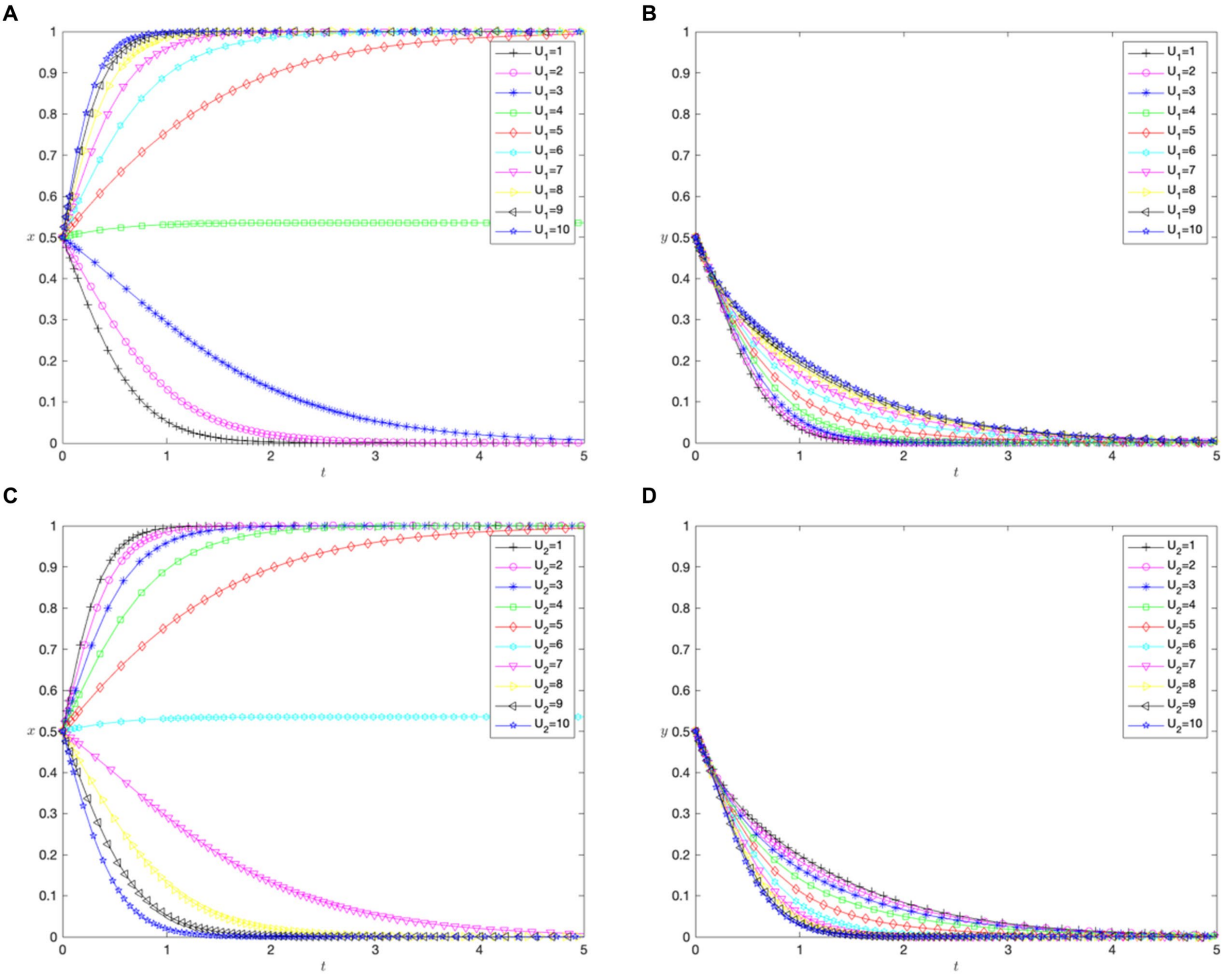
The impact of patient medical utility. **(A)** Impact of the utility of cross-regional treatment ( $U_{1}$ ) on patients. **(B)** Impact of the utility of cross-regional treatment ( $U_{1}$ ) on hospitals. **(C)** Impact of the utility of in-area medical care ( $U_{2}$ ) on patients. **(D)** Impact of the utility of in-area medical care ( $U_{2}$ ) on hospitals.

Figures 3A,B show the impact of the utility coefficient for patients’ cross-regional treatment on their choice of treatment location and the hospital cross-regional settlement policy. When the utility coefficient $U_{1}$ is low, patients with malignant tumors typically opt for medical care within their region. However, as $U_{1}$ increases to five, their evolutionarily stable strategy shifts toward cross-regional treatment due to heightened utility, making treatment in other areas more advantageous. Irrespective of the variations in $U_{1}$ , hospitals determine that the optimal strategy is not to implement a cross-regional settlement policy.

Figures 3C,D show the impact of the utility coefficient for in-area medical care ( $U_{2}$ ) on patients’ choice of treatment location and cross-regional hospital settlement policies. In contrast to the utility coefficient for cross-regional treatment ( $U_{1}$ ), a lower $U_{2}$ value indicates that patients with malignant tumors prefer cross-regional treatment. When $U_{2}$ increased to seven, the evolutionarily stable strategy shifted to in-area medical care. This suggests that increases in the utility of in-area care influence patients’ choices more sensitively than those of cross-regional care. Similar to the effects of $U_{1}$ , regardless of the variations in $U_{2}$ , the optimal strategy for hospitals is not to implement a direct settlement policy for cross-regional treatment.

#### 4.2.2 The impact of patient medical costs

**Figure 4:**
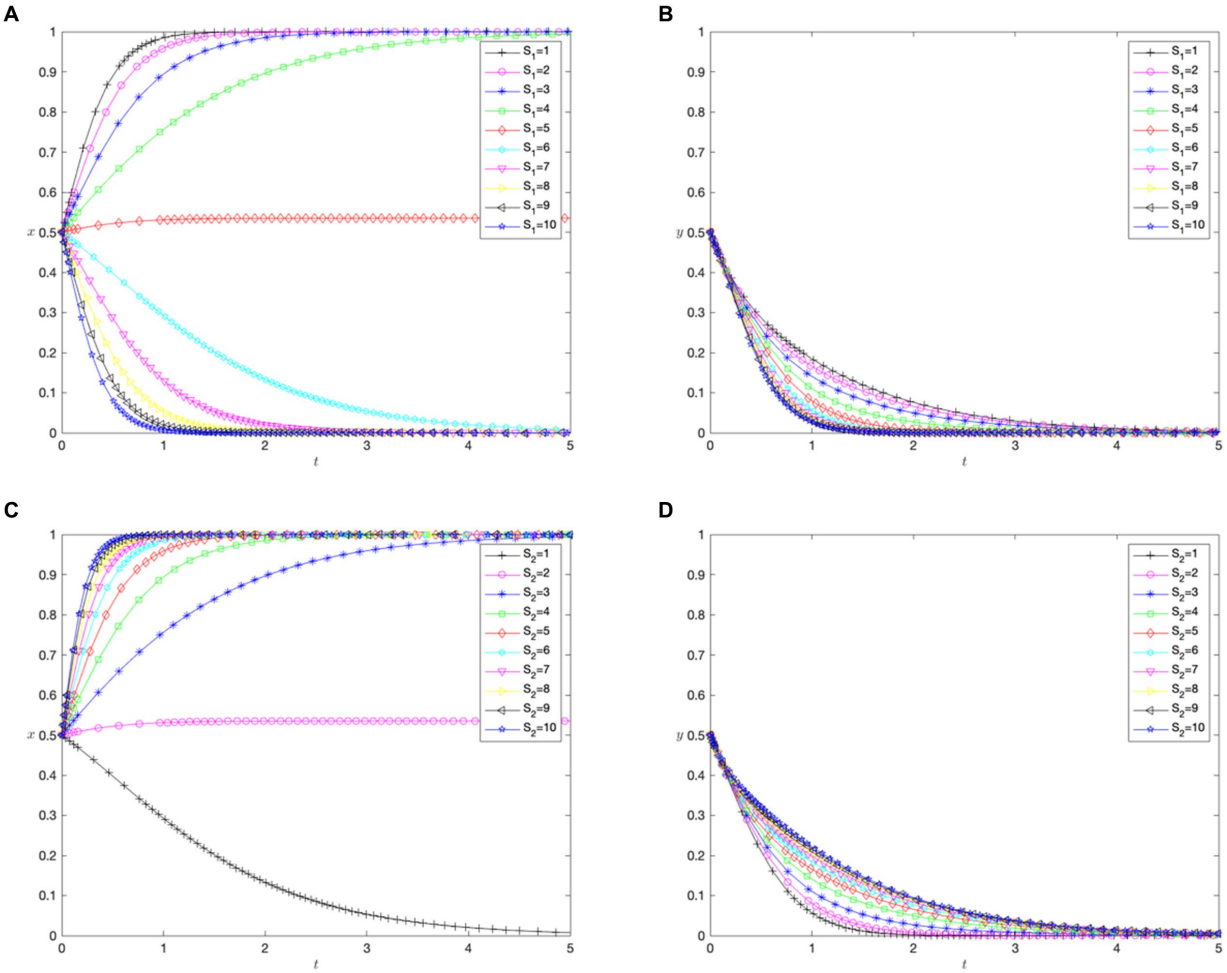
The impact of patient medical costs. **(A)** Impact of medical costs paid by patients after cross-regional treatment ( $S_{1}$ ) on patients. **(B)** Impact of medical costs paid by patients after cross-regional treatment ( $S_{1}$ ) on hospitals. **(C)** Impact of medical costs paid by patients after in-area medical care ( $S_{2}$ ) on patients. **(D)** Impact of medical costs paid by patients after in-area medical care ( $S_{2}$ ) hospitals.

Figures 4A,B demonstrate how the coefficient of medical costs paid by patients for cross-regional treatment ( $S_{1}$ ) affects the patients’ choice of treatment location and hospital cross-regional settlement policies. With a lower $S_{1}$ , patients with malignant tumors favor cross-regional treatment. As $S_{1}$ increased to 6, their preference shifted to in-area medical care. However, $S_{1}$ did not impact hospitals’ settlement policies, and not implementing direct settlement remained the best strategy.

Figures 4C,D demonstrate how the coefficient of the medical costs paid by patients for in-area treatment ( $S_{2}$ ) affected their choices. When $S_{2}$ is 1, the patients opt for in-area medical care. As $S_{2}$ increases, they lean toward cross-regional treatment. The rate of change in $S_{2}$ compared with $S_{1}$ suggests a higher sensitivity to in-area treatment costs. Despite varying $S_{2}$ , hospitals chose not to implement direct cross-regional settlements.

#### 4.2.3 The impact of other costs of cross-regional treatment for patients

**Figure 5:**
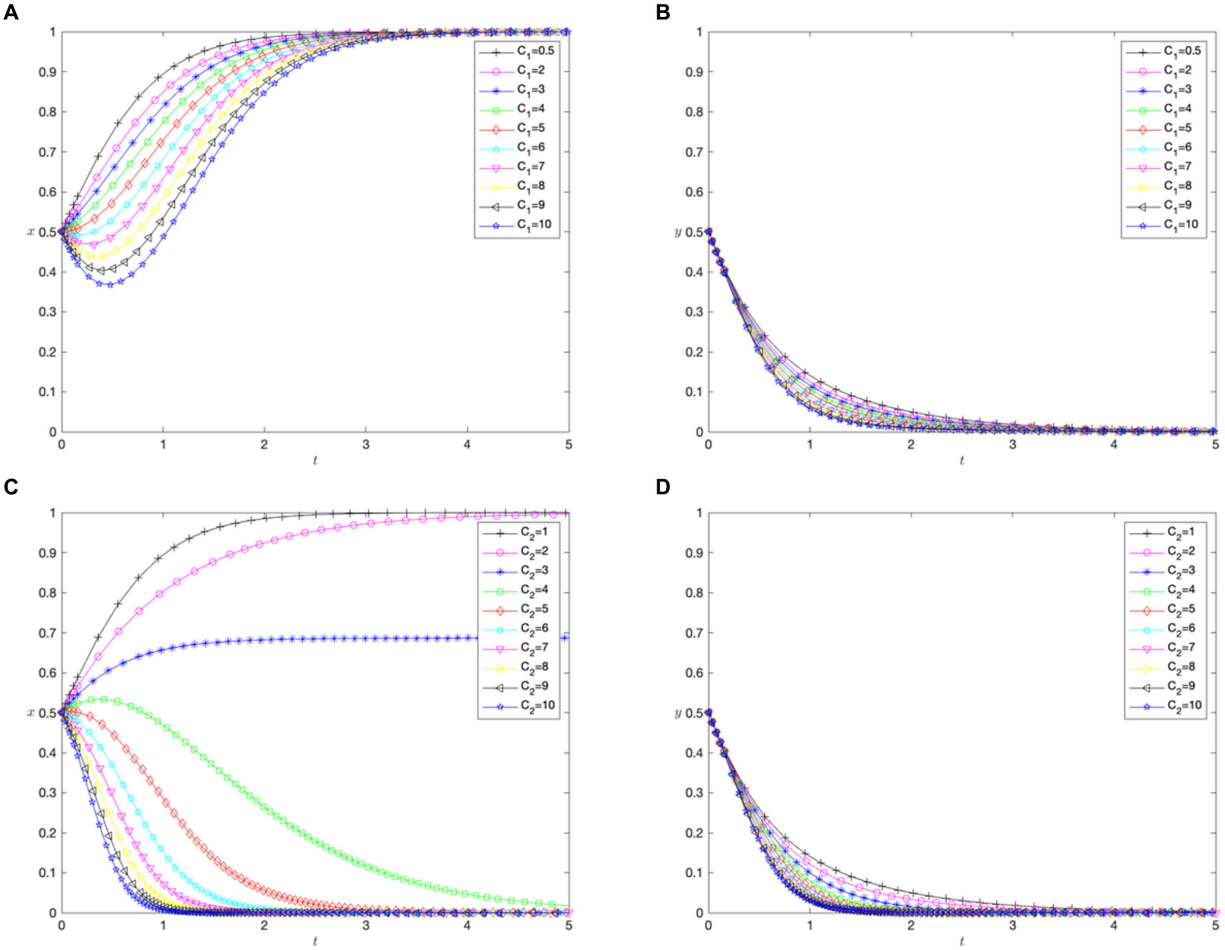
The impact of other costs of cross-regional treatment for patients. **(A)** Impact of other costs of cross-regional treatment with direct settlement ( $C_{1}$ ) on patients. **(B)** Impact of other costs of cross-regional treatment with direct settlement ( $C_{1}$ ) on hospitals. **(C)** Impact of other costs of cross-regional treatment without direct settlement ( $C_{2}$ ) on patients. **(D)** Impact of other costs of cross-regional treatment without direct settlement ( $C_{2}$ ) on hospitals.

Figures 5A,B highlight the impact of the coefficient of other costs under direct settlement ( $C_{1}$ ) on the choice of treatment location by patients and the cross-regional settlement policies of hospitals. As the value of $C_{1}$ increased, patients consistently chose cross-regional treatment, and hospitals opt not to settle directly for cross-regional treatments.

Figures 5C,D highlight the effect of the coefficient of other costs without direct settlement ( $C_{2}$ ) for cross-regional treatment on similar choices. When $C_{2}$ ranged from 1 to 3, patients were more likely to choose cross-regional treatment. However, as $C_{2}$ increased, they switched to in-area medical care. In response to changes in $C_{2}$ , hospitals maintain a strategy of not directly settling for cross-regional treatment.

#### 4.2.4 The impact of hospital benefits from cross-regional treatment

**Figure 6:**
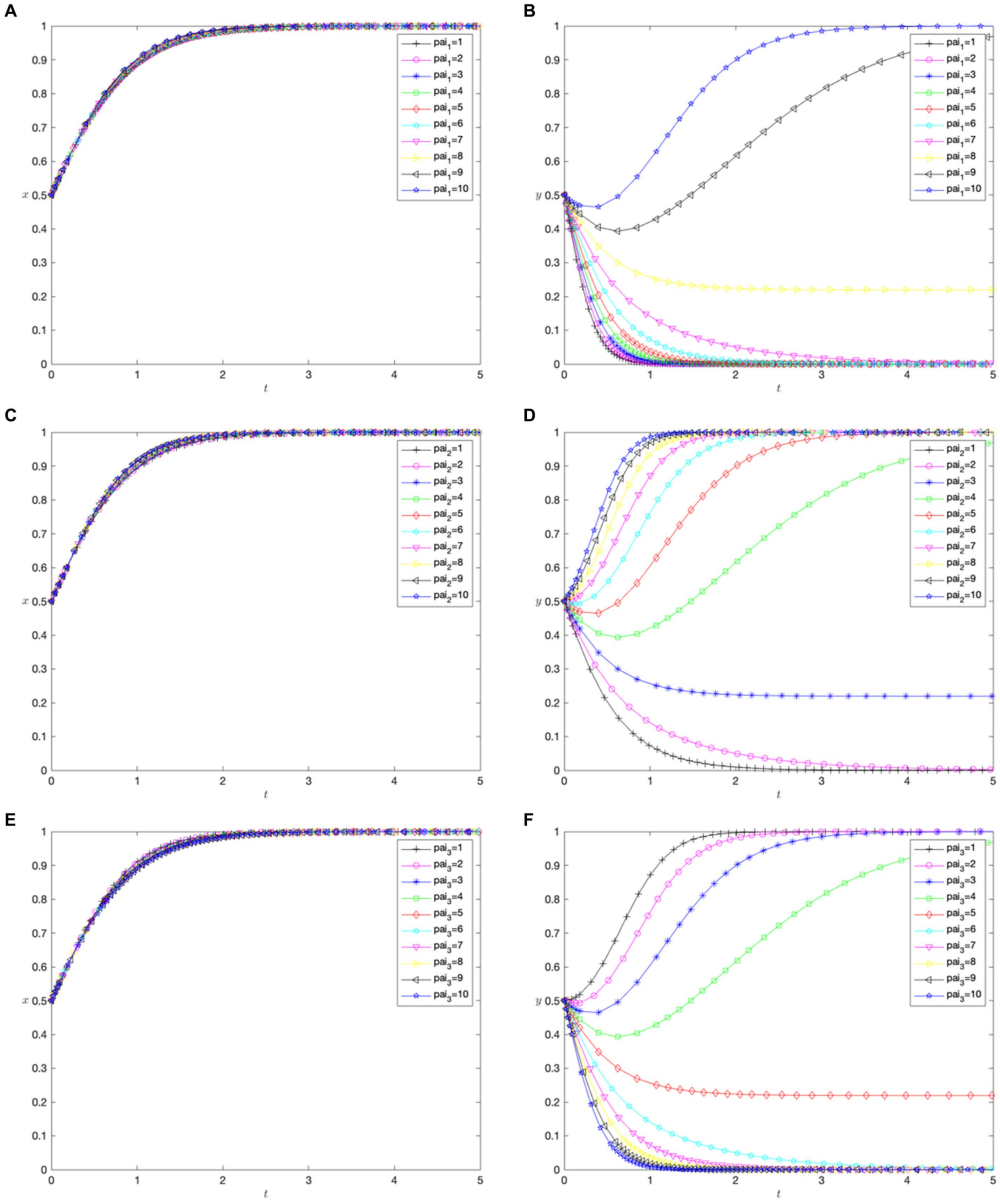
The impact of hospital revenues from cross-regional treatment. **(A)** Impact of financial benefits of implementing direct settlement for cross-regional treatment ( $\pi_{1}$ ) on patients. **(B)** Impact of financial benefits of implementing direct settlement for cross-regional treatment ( $\pi_{1}$ ) on hospitals. **(C)** Impact of technological advancement benefits of implementing direct settlement for cross-regional treatment ( $\pi_{2}$ ) on patients. **(D)** Impact of technological advancement benefits of implementing direct settlement for cross-regional treatment ( $\pi_{2}$ ) on hospitals. **(E)** Impact of financial benefits of implementing direct settlement for local treatment ( $\pi_{3}$ ) on patients. **(F)** Impact of financial benefits of implementing direct settlement for local treatment ( $\pi_{3}$ ) on hospitals.

Figures 6A,B reveal how the financial benefit coefficient ( $\pi_{1}$ ) for hospitals offering cross-regional treatment affects patients’ choice of treatment location and hospital settlement policies. As $\pi_{1}$ increases, patients consistently choose cross-regional treatment, indicating that hospitals’ financial gains from such treatment do not affect their choices. However, hospitals tended to shift from implementing a direct settlement policy to implementing it.

Figures 6C,D reveal how the technological advancement benefit coefficient ( $\pi_{2}$ ) for hospitals affects choices. An increase in $\pi_{2}$ leads to patients’ consistent preference for cross-regional treatment, showing that hospitals’ technological advancement benefits from cross-regional treatment do not affect patients’ choices. However, hospitals tended to shift from implementing the direct settlement policy to not implementing it, with a higher rate of change than $\pi_{1}$ , indicating a more substantial impact on hospital policies.

Figures 6E,F reveal how the financial benefit coefficient ( $\pi_{3}$ ) for hospitals not implementing direct settlement affects choices. Increasing $\pi_{3}$ results in patients always choosing cross-regional treatment, suggesting that hospitals’ technological advancement benefits do not influence patients’ choices. However, hospitals’ strategies changed from implementing to not implementing the direct settlement policy, opposite to the trend seen with $\pi_{1}$ .

#### 4.2.5 The impact of hospital costs for cross-regional treatment

**Figure 7:**
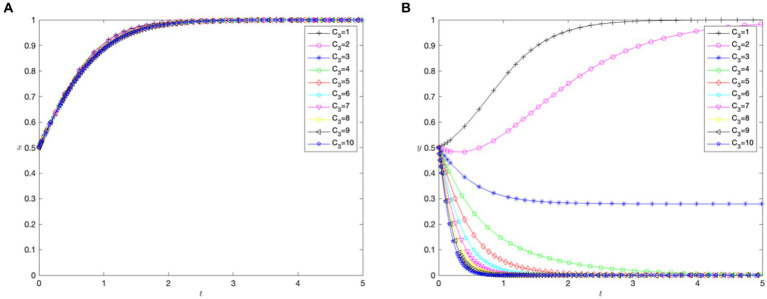
The impact of hospital costs for cross-regional treatment. **(A)** Impact of input costs of implementing direct settlement for cross-regional treatment ( $C_{3}$ ) on patients. **(B)** Impact of input costs of implementing direct settlement for cross-regional treatment ( $C_{3}$ ) on hospitals.

Figures 7A,B depict the impact of the input cost coefficient ( $C_{3}$ ) for hospitals implementing direct settlement in cross-regional treatment on patients’ choice of treatment location and hospital settlement policies. When $C_{3}$ increases, the patients opt for cross-regional treatment, indicating that hospital operational costs did not directly affect their choices. Hospitals are more likely to implement direct settlement when $C_{3}$ is low but become reluctant when $C_{3}$ rises above 4.

## 5 Discussion and implications

### 5.1 Discussion

Driven by the pursuit of high-quality medical resources and additional considerations, patients with malignant tumors require cross-regional treatment; however, hospitals lack the motivation to implement direct settlement policies. The evolutionary game model in this study has seven scenarios, with four involving patients choosing cross-regional treatment and only two in which hospitals opt for direct settlement. The simulation analysis shows that the evolutionary game results correspond to Scenario 6, described earlier, where the optimal strategy for patients is cross-regional treatment, whereas it does not implement direct settlement for hospitals. Regardless of changes in the patient’s utility ( $U_{1}$ and $U_{2}$ ), medical costs ( $S_{1}$ and $S_{2}$ ), and other costs of the patient’s cross-location medical treatment ( $C_{1}$ and $C_{2}$ ), the hospital’s gaming strategy did not change. This indicates that whether the hospital executes a direct settlement policy is not affected by the patient’s benefit in the game between the two sides. In addition, the two parties only cooperate in the seventh scenario in the stability analysis; that is, the patient chooses to seek cross-regional treatment and the hospital implements a direct settlement policy. The conditions for realizing this scenario were $C_{1}+S_{1}-S_{2}-U_{1}+U_{2}<0\text{,}$ $C_{3}-\pi_{1}-\pi_{2}+\pi_{3}<0$ , and $S_{2}-S_{1}+U_{1}-U_{2}-C_{2}>0$ . However, meeting these conditions is challenging, and the lack of effective incentives for hospitals to implement direct settlement policies explains why the current cross-regional treatment policies are not as widespread as anticipated (11).

The key factors influencing the willingness of some patients with malignant tumors include the costs of in-area medical care, costs of cross-regional treatment without direct settlement, and the utility of cross-regional treatment. A comparison of the impact of various parameters on the evolutionary outcome reveals that parameters with fast convergence rates and short convergence times included the medical costs paid by patients after in-area medical care ( $S_{2}$ ), other costs of cross-regional treatment without direct settlement ( $C_{2}$ ), and the utility of cross-regional treatment ( $U_{1}$ ). First, Patients with malignant tumors are sensitive to changes in in-area medical care costs because their medical expenses are high. Between 2012 and 2014, the average medical expenses for these patients in China were approximately $ 10,000 (59), whereas the average medical expenses for general patients were approximately $ 1,000 (60). Thus, medical expenses for patients with malignant tumors are significantly high. The cross-regional settlement follows the principle of “insurance catalog of the treatment location and reimbursement standards of the insured site.” Under the same treatment standards, some patients often receive more medical service reimbursements, such as reimbursements for targeted drugs, in cross-regional hospitals with sufficient medical insurance coverage, such as reimbursements for targeted drugs (27). Under settlements based on the medical insurance catalog of the treatment location, actual expenses are often lower than those for in-area treatment (23). Furthermore, patients with malignant tumors are highly sensitive to the indirect settlement costs associated with cross-regional treatment. This is because their condition requires long-term and extensive treatment, with a propensity for recurrence or metastasis, necessitating frequent diagnosis, treatment, and follow-up (61). Factors such as travel and accommodation expenses, as well as efforts during cross-regional medical visits greatly influence treatment decisions (62). The absence of direct insurance settlements in cross-regional hospitals further increases these costs. Moreover, patients are sensitive to cross-regional treatment owing to the high mortality rate associated with malignant tumors. In China, the five-year relative survival rate of patients with malignant tumors is approximately 40.5% (16). Therefore, patients tend to seek medical care to achieve better treatment outcomes.

Therefore, patients with malignant tumors could adopt the following strategies. From the perspective of medical utility ( $U_{1}\;\mathrm{and}\;U_{2}$ ), patients with high mortality and low mortality tumors should have different strategies. Patients with high mortality cancers such as pancreatic cancer, liver cancer, lung cancer, and esophageal cancer (63) should consider traveling to regions with more developed medical resources to get better five-year survival rates. For patients with low mortality cancers, such as breast cancer, prostate cancer, thyroid cancer, and melanoma (63), the medical utility of local treatment is similar to that of treatment in other regions due to mature treatment methods and standardized diagnostic and treatment guidelines. And staying local for treatment can also save on the additional costs associated with cross-regional treatment. Regarding medical costs( $S_{1}\;\mathrm{and}\;S_{2}$ ), the treatment expenses for patients in the surgical phase are significantly lower than for those requiring chemotherapy, targeted therapy, or immunotherapy (64, 65), thus local treatment is advisable for patients in the surgical phase. Non-surgical patients, in search of better health insurance reimbursement policies, might choose to travel to regions with a broader coverage of health insurance. Considering the indirect costs of cross-regional treatment without direct settlement ( $C_{2}$ ), patients with malignant tumors who do not require immediate surgery or emergency care could use telemedicine services, such as video consultations or telephone medical advice, to minimize the need for travel.

Hospitals’ motivations to implement direct settlement policies are influenced by two main factors: technological advancement benefits and input costs. A comparison of the impact of different parameters shows that the parameters with fast convergence rates and short convergence times include the technological advancement benefits of implementing direct settlement for cross-regional treatment ( $\pi_{2}$ ), and the input costs of implementing direct settlement for cross-regional treatment ( $C_{3}$ ). The simulation analysis confirms that the convergence of technological advancement benefits for hospitals is faster than that of their financial benefits. This can be attributed to the shift in focus of China’s public hospitals toward high-quality and substantive development, with sustainable operational capabilities and the construction of clinical specialties becoming key priorities in hospital development (66). This aligns with the direct settlement policy for cross-regional treatment as it provides hospitals with more elements of demand from a resource allocation perspective. This policy enables hospitals to identify patients with urgent treatment needs and higher disease complexity for diagnosis and treatment (67). However, before implementing a cross-regional treatment policy, hospitals must revamp their information systems and ensure sufficient operational costs for patient policy education, medical insurance reimbursements, and analysis. Consequently, the input costs for hospitals in direct settlements for cross-regional treatment are relatively high, which affects their willingness to implement this policy.

Therefore, hospitals could employ several strategies. First, based on their financial health, hospitals should rigorously assess the investment costs associated with implementing direct settlement systems ( $C_{3}$ ), which includes expenses related to upgrading information systems, patient education, and handling of medical insurance reimbursements. For financially robust and well-staffed hospitals, it is advisable to seek qualification for direct settlements, facilitating more convenient services for patients undergoing treatment for malignant tumors from different regions. Conversely, hospitals with constrained financial resources should prioritize the care of potential malignant tumor patients within their jurisdiction. Secondly, considering the advantages of technological advancements ( $\pi_{2}$ ) and patient medical utility ( $U_{1}\;\mathrm{and}\;U_{2}$ ), tertiary regional hospitals should invest in the latest medical equipment and technologies, such as PET-CT scanners and advanced radiation therapy devices, to provide precise diagnostics and treatments. These hospitals should also establish multidisciplinary treatment teams to manage patient care comprehensively and prioritize the creation of day clinics and treatment centers to enable patients from various locations to receive radiotherapy and chemotherapy in the shortest possible time (68). Other hospitals may establish cooperative medical networks or offer telemedicine services, leveraging regional hospitals’ expertise and diagnostic capabilities to provide necessary treatments and consultations. Finally, all hospitals should optimize internal diagnostics, treatments, examinations, and report delivery processes, reducing waiting times for patients with malignant tumors. This optimization helps to mitigate the additional costs associated with medical appointments, such as expenses for offsite accommodations, family caregiving, and loss of income due to work interruptions.

### 5.2 Theoretical implications

We developed a behavioral analysis framework that classifies patients with malignant tumors and hospitals into two groups, offering theoretical validation for actual scenarios. In dissecting the treatment-seeking behavior associated with cross-regional settlement methods, we validated the urgent medical needs of cross-regional patients and the limited motivation of hospitals for cross-regional direct settlement. We identified the key factors that enhance cooperation between the two parties, thereby validating and clarifying the realities of cross-regional treatment in China. The analytical framework intentionally omits the impact of existing policies, which paradoxically lends theoretical support to the development of new direct settlement policies for cross-regional medical treatment.

Additionally, the incorporation of game theory into our research methodology has significantly augmented the analytical depth of studies on cross-regional treatment. Confronted with a scarcity of empirical data, our reliance on game-theoretic paradigms amplifies the interpretative robustness of our findings. The bounded rationality inherent in the Evolutionary Game Theory is more congruent with the real-world dynamics of cross-regional treatment and the execution of hospital policies. Furthermore, the dynamic development perspective of the Evolutionary Game Theory compensates for potential short-term strategic behaviors between patients and hospitals in cross-regional contexts. This method led to a more accurate and comprehensive exploration of interactions between the two groups.

Moreover, this study, which is centered on the Chinese context, injects novel perspectives into global research. With the burgeoning demand for cross-regional treatment worldwide (69), as evidenced in nations such as the United States (70), the EU (71), Russia (72), and India (73), our study enriches scholarly research on China’s cross-regional treatment dynamics. It can provide valuable insights on a global scale for regions grappling with the limitations of geographic coverage in medical insurance coordination, inadequate health insurance reimbursement for non-local treatments, and the high demand for cross-regional medical services among patients with malignant tumors. For example, in Brazil, despite the government’s promotion of fluvial mobile units, the remote Amazon region still faces severe shortages in medical services and insurance coverage (74); in the Philippines, although the PhilHealth insurance system is in place, patients in provinces outside the capital region, especially on the more distant islands, often face medical expenses that exceed insurance coverage (75); in Italy, particularly between the north and the south, the uneven distribution of medical resources leads many cancer patients from the southern regions to seek more advanced treatments in northern cities like Milan or Turin (76); in Thailand and Cuba, the thriving health tourism sector, known for quality and affordability, draws international patients for advanced treatments like cardiac and cosmetic surgeries (77).

### 5.3 Policy implications

To promote a risk-sharing mechanism between the treatment location and the insurance registration location, and to ensure that tertiary hospitals offer direct settlement services. Local health insurance management departments should proactively implement policies for offsite medical treatment and direct settlement, incorporating a risk-sharing mechanism between the treatment and insurance registration locations to share the excess medical costs incurred by patients seeking offsite treatment, thus incentivizing the insurance department at the treatment location to enforce regulations. Moreover, with the promotion of direct settlement policies, it is essential to consider the disparities in medical service accessibility across various provinces and cities caused by the household registration system. High-level tertiary hospitals should take the initiative to qualify for offsite medical treatment direct settlement, whereas weaker first- and second-level hospitals should be phased out gradually. This ensures that patients from different locations receive the appropriate treatment at high-level hospitals.

Moreover, by enhancing the provincial level of medical insurance pooling and exploring the adoption of the Diagnosis-Related Group (DRG) and Diagnosis Intervention Packet (DIP), strides can be made to augment the healthcare system’s effectiveness. Achieving nationwide health insurance coordination within a short timeframe is not realistic because of disparities in the three-level catalogs and drug codes across different regions in China. Therefore, the primary focus should be gradually improving provincial health insurance coordination and establishing consistent settlement standards for medical treatments within the same province. Besides, due to the current fee-for-service payment model adopted for medical insurance in cross-regional medical treatments, hospitals are more inclined to admit patients from other regions, resulting in disparities in healthcare access between local and non-local patients (12). To address this issue, it is proposed to initiate reforms in hospitalization expense management by implementing a trial of both the DRG and DIP with monthly pre-settlement and annual clearing modes can be introduced. This strategy will incentivize hospitals to make more considered diagnoses and treatment decisions, enhance equity in access to medical resources, and ultimately improve the overall quality of healthcare services.

Using digital health technologies to significantly enhance remote initial diagnostic capabilities and promotes collaborative diagnostics and treatments across healthcare regions. Through internet-based medical platforms, physicians can provide remote initial consultations for malignant tumor patients seeking care outside their local facilities. These consultations facilitate preliminary symptom assessment and guide patients on whether they should seek further examinations or treatments at higher-level hospitals. Additionally, these technologies are instrumental in implementing post-treatment follow-ups and continuous health monitoring. This approach not only alleviates unnecessary patient travel and financial expenditures but also significantly reduces the wastage of medical resources. Simultaneously, the establishment of cross-regional medical consortia is imperative (78). Such consortia facilitate the sharing of medical records among hospitals across different regions and foster professional discussions and collaborations, ensuring that patients receive standardized, high-quality diagnostic and treatment services even when away from their insured location.

It is important to note that the implementation of these strategies varies among different regions and hospitals, with some areas facing greater challenges due to economic, resource, or management disparities. This interplay between regions and hospitals can impact the uniform implementation and effectiveness of policies, necessitating more customized and regional strategies to address these challenges.

### 5.4 Limitations and future work

This study enriches the academic discussion on cross-regional medical treatment from a Chinese perspective, showing significant theoretical value. However, there are several noteworthy limitations in the study. Firstly, due to challenges in obtaining empirical data and insufficient funding, this study uses Evolutionary Game Theory to theoretically explore the interaction strategies between the medical behaviors of patients with malignant tumors and settlements in some hospitals. This approach is close to reality but not entirely based on actual situations. Furthermore, the analysis based on the technically complex Evolutionary Game Theory is difficult for the general readership to understand. Therefore, future research could, on the one hand, propose new hypotheses based on the results of this model, collect empirical data for analysis to enhance the real-world applicability of the research; on the other hand, conduct case studies to deeply explain the conclusions of the evolutionary game model, enhance the implementation of policy recommendations, and improve understanding among policymakers and practitioners. Secondly, the model used in this study simplifies and abstracts the complex interaction system between patients with malignant tumors and some hospitals, providing valuable insights into the balance of interests between hospitals and patients. However, it fails to capture the complexity of real-world issues fully, which may lead to overly generalized conclusions. For example, it does not consider differences in hospital levels or the subtle differences in direct settlement policies across different provinces. Future research should explore these factors to deepen understanding of the issue. Thirdly, the COVID-19 pandemic disrupted the provision and access to healthcare services (79). Although retrospective studies on the cross-regional settlement of patients with malignant tumors may seem less relevant in the post-pandemic era from today’s perspective, such research is still extremely valuable from the perspective of medical service fairness. It not only helps to understand how the pandemic has exacerbated medical inequalities for specific groups but also provides an essential basis for formulating fairer and more effective health policies in potential future crises.

## 6 Conclusion

Cross-regional medical treatment has become necessary in response to the insufficient distribution of high-quality medical resources. The localized management of medical insurance in China is actively improving the accessibility and convenience of medical services through direct settlement services. Patients with malignant tumors, the leading group seeking cross-regional treatment, face significant financial burdens. Based on this, this study presents an evolutionary game model between patients with malignant tumors and some hospitals, analyzing the optimal strategies, stability of strategy combinations, and interrelations of influencing factors. This theoretical framework provides a deeper understanding of this issue. This study proposes potential measures to draw further attention to the issue of cross-regional medical settlements in China.

## Data availability statement

The raw data supporting the conclusions of this article will be made available by the authors, without undue reservation.

## Author contributions

XZ: Conceptualization, Methodology, Software, Writing – original draft, Writing – review & editing. LL: Writing – review & editing. DZ: Conceptualization, Methodology, Supervision, Writing – review & editing.
